# Exploring the Genetic Conception of Obesity via the Dual Role of FoxO

**DOI:** 10.3390/ijms22063179

**Published:** 2021-03-20

**Authors:** Tapan Behl, Ishnoor Kaur, Aayush Sehgal, Sukhbir Singh, Gokhan Zengin, Nicoleta Negrut, Delia Carmen Nistor-Cseppento, Flavia Maria Pavel, Raluca Anca Corb Aron, Simona Bungau

**Affiliations:** 1 Department of Pharmacology, Chitkara College of Pharmacy, Chitkara University, Punjab 140401, India; ishnoorkaur7@gmail.com (I.K.); aayushsehgal00@gmail.com (A.S.); singh.sukhbir12@gmail.com (S.S.); 2 Department of Biology, Faculty of Science, Selcuk University Campus, Konya 42130, Turkey; biyologzengin@gmail.com; 3 Department of Psycho-Neuroscience and Recovery, Faculty of Medicine and Pharmacy, University of Oradea, 410073 Oradea, Romania; lnm_n10@yahoo.com (N.N.); delia_cseppento@yahoo.com (D.C.N.-C.); 4 Department of Preclinical Disciplines, Faculty of Medicine and Pharmacy, University of Oradea, 410073 Oradea, Romania; flavia.bontze@gmail.com (F.M.P.); raluca14@yahoo.com (R.A.C.A.); 5 Department of Pharmacy, Faculty of Medicine and Pharmacy, University of Oradea, 410028 Oradea, Romania

**Keywords:** obesity, metabolic syndromes, FoxO, AKT pathway, oxidative stress, insulin

## Abstract

Obesity or overweight are not superficial problems, constituting a pressing issue. The obesity index has almost tripled since 1975, which is an alarming state. Most of the individuals are currently becoming overweight or have inappropriate body mass index (BMI) conditions. Obesity is characterized by increased fat accumulation and thus poses a higher health risk. There is increased size and volume of fat cells in the body, which usually accounts for obesity. Many investigations have been carried out in this area, such as behavioral improvements, dietary changes, chemical involvements, etc., but presently no such goals are established to manage these health concerns. Based on previous literature reports and our interpretation, the current review indicates the involvement of various transcriptional and transporter functions in modifying the above-mentioned health conditions. Various transcriptional factors such as Forkhead box O1 (FoxO1) impart a significant effect on the physiology and pathology of metabolic dysfunction such as obesity. FoxO1 plays a dual role whether in the progression or suppression of metabolic processes depending on its targets. Thus, in the current study, will be discussed the dual role of FoxO1 in metabolic conditions (such as obesity), also summarizing the role of various other transcriptional factors involved in obesity.

## 1. Introduction

Obesity accounts for a body mass index (BMI) of over 30 kg/m^2^. The worldwide frequency/rate of obesity was estimated to escalate to 1.9 billion adults and 340 million adolescents/children between 5 and 19 years old in 2016 [1]. Additionally, obesity and overweight might elevate the risk of dementia with age [2]. The role of overweight and obesity in inducing cognitive impairment or dementia is therefore critical to health and may be linked with higher fetch. Future research on the role of BMI in memory loss has not yet provided a definitive picture. Certain studies have shown no correlation or even reduced BMI with dementia or Alzheimer’s disease (AD) [3,4], whereas others have indicated higher BMI as a possible cause for dementia [5], or overweight to be correlated with dementia decades later. At least partially, the dynamic interplay may explain the various findings obtained by different experiments.

Many experiments suggested that impaired metabolism in obesity, overweight, or type 2 diabetes mellitus (T2DM) influences the physiological actions of the brain, such as memory and cognitive function [6,7]. Adipose tissue regulates metabolism and energy balance throughout the body and is a significant targeted therapy for metabolic syndrome.

There is intensive research related to the differentiation and role of white adipocytes [8], capable of storing and releasing lipids as well as releasing hormones (adipokines), and brown adipocytes capable of burning off energies [9,10]. Forkhead Box O1 (FoxO1), a participant of the FoxO family of transcription factors, plays a significant role in both the adipocyte forms. FoxO1 modulates the transcription of downward streaming metabolic regulation facilitating genes. Dysregulation of the Fox1 pathway contributes to many metabolic conditions, especially obesity [11,12]. The FoxO transcription factors are essential moderators of insulin and insulin-like growth factor 1 (IGF-1)-mediated impact. These transcription factors include neuronal proliferation, differentiation, stress response and detoxification by β-amyloids [13,14].

More than 100 variants of this gene family have been found since the very first investigation of the *Drosophila melanogaster* fly forkhead gene, and 19 living subsets have now been proven to persist [15,16]. The FoxO protein is the subtype of transcription factor (TF) marked by the N-terminal edge of the gene of a retained deoxyribonucleic acid (DNA)-binding interface (the Forkhead box) [17,18]. In response to outer signals [19], FoxO links the consensus DNA-binding factor inside the promoter of its target genes and modulates their transcription.

FoxO1, FoxO3a, FoxO4, and FoxO6 are the major transcription factors which constitute the “O” subset of the FOX family. FoxO2 is known as a FoxO3 homolog and FoxO5 is present mostly in Danio rerio (FoxO3b) [20,21]. They are widely present in living organisms and play a crucial role in invertebrates, mammalian survival, the suppression of tumor proliferation, energy metabolism control and cellular response initiation [22]. Various growth factors, including insulin-like growth factor, neurotrophins, cytokines, insulin, and triggers of oxidative damage, reflect fluctuations in the function of FoxO via signal transduction, involving a wide range of kinases and other molecules [23,24]. FoxO1 is shown to be a prominent and extensive unit of the FoxO community, which exhibits key regulatory functions in the transcription process [25]. The FoxO1 gene is located on chromosome 13 and is responsible for FoxO1 protein synthesis. FoxO1 comprises four functional regions, namely the region of the nuclear export signal (NES), nuclear localization signal (NLS), the region of trans-activation, and the region of the forkhead [17,26]. For its regulatory influence, Fox is an abundant region, which is made up of triplet β-strands (S1, S2, and S3), two-winged loops (W1, W2), and four helices (H1, H2, H3, and H4). Amongst them, H3 is linked to the DNA strand and, because of its precise base-centered contact ability and exposed surface with the genetic sequence 5′-TAAGTCA-3′, it acts as a DNA recognition element. Crystal structure research suggests that FoxO1 links two consecutive tri-nucleotide sequences, namely 5′-GTAAA(T/C) AA-3′, which are classified as the 5′-(C/A) (A/C) AAA(C/T) AA-3′and Daf-16 binding element (DBE), which is responsible for the translation of the insulin-responsive element (IRE) coding [27,28]. In contrast, W1, S2, and S3 are responsible for the stabilization of the H3 protein-DNA composite to create a link to DNA phosphate bonding [29].

The origin of the control of FoxO1 action is the complex interaction domains with downward streaming DNA. FoxO1 mediates different targets, including such proteins indulged in programmed cell death as well as self-eating, anti-oxidative enzymes, genes for cell cycle disruption [30,31] and metabolic and immune regulators, further making it a complex activity component for transcriptional processes. In contrast, the translation of FoxO1 or the link to the FoxO1 gene affects many aspects of upward streaming elements to entail signaling pathways, which influence the transcriptional role of FoxO1 in several main biological processes [32,33]. Due to its wide genetic expression, FoxO1 preserves cellular homeostasis and changes in response to different stimuli, which may be a critical target for the prevention and treatment of the disease. The post-transcriptional alterations affect the physiological relevance of FoxO1 in diseases which are still unclear and also contradictory at some stages.

Thus, we have tried to explain the potential modulation and function of FoxO1 in the development of many disorders [34,35]. This paper summarizes the FoxO1 control mechanism behind the disease as well as other metabolic diseases linked to it.

## 2. FoxO1: An Effective Tactic to Combat Obesity

Fat cells are widely known as the central moderators of homeostasis of the whole-body energy and are a prime targeted therapy for metabolic syndrome as a consequence. Thus, there is extensive study of the differentiation and functioning of white fat cells, which are responsible for the synthesis, storage, and release of lipids, adipokines, and brown adipocytes, which can burn off adenosine triphosphates (ATPs) [36,37]. For both categories of adipocytes, FoxO1, a participant of the FoxO transcription factors, exhibits a key role.

When a high fat diet (HFD) is administered, FoxO1+/− mice with smaller, “healthier” fat cells are protected from T2DM, likely by the suppression of the peroxisome proliferator-activated receptor (PPAR)-γ1 adipocytic transcriptional elements [38,39]. A higher-negative side of FoxO1 increases ATP expenditure in vivo in brown adipose tissue, implying that FoxO1 inhibition in white and/or brown fat cells can pose an appealing treatment target to maintain body homeostasis [40]. These divisions of the cell cycle pose problems in a series of in vitro experiments. Zou et al. showed that FoxO1 cannot phosphorylate adipocytes, which are inversely linked to its function, giving a composite, tide-like sequence throughout adipocyte differentiation based upon the timing of the addition and blockade of FoxO1 with the specific inhibitor, AS1842856, which can resist further transcriptional events [41].

### 2.1. FoxO1 Role in Normal Physiology and Pathogenesis of Metabolic Disorders

Earlier investigations have reported that FoxO1 regulates downstream processes of gene expression, including [42] anti-oxidative enzymatic distress, [43] cell cycle hindered genes, [44] genes linked with apoptosis, and self-eating, [45] metabolic and immune controllers. FoxO1 and these earmarks thereby form signaling associated pathways involved in antioxidant distress, cell viability, cellular death, hindrance of the cell cycle, self-eating, metabolic control, and immunity [46]. The expression of various earmarks is consistent with FoxO1 action, causing direct or indirect different physiologic and pathologic modifications. Nonetheless, when positioned in the cytoplasm [47], FoxO1 undergoes trapping and cannot further regulate its downward stream earmarks. Zhao et al. reported the significance of post-translational modifications of proteins belonging to the FoxO family (such as ubiquitination, acetylation, phosphorylation, glycosylation and methylation), which occur as a result of cellular stress. These modifications aid the regulation of FoxO protein subcellular localization, along with their deoxyribonucleic acid (DNA) binding, half-life, and interaction capability with other cellular proteins and transcriptional activity [48].

### 2.2. FoxO1: Activity and Expression Regulation

Acetylation and deacetylation, by controlling the trans-activation of transcription elements, are accountable for the consistent regulation of ATPs’ metabolism. The two categories of enzymes, histone deacetylases (HDACs) and histone acetyltransferases (HATs) [47,49], regulate protein acetylation at lysine residues. HATs transfer acetyl groups to lysine residues in target proteins, whereas HDACs catalyze the reverse reaction rate [50]. Acetylation and deacetylation closely control the transcriptional operation of FoxO1. Furthermore, FoxO1 deacetylation facilitates the nuclear storage of FoxO1, and thus increases its output [51]. In brief, the most significant signaling pathways influencing FoxO1 expression levels are acetylation and deacetylation, along with the phosphorylation of FoxO1, which significantly affects its protein levels. Its acetylation condition, which affects its subcellular localization, regulates the physiological functions of FoxO1 (its expression being controlled by co-activator, P300) [52].

Mutations that imitate the acetylated condition make FoxO1 more susceptible to phosphorylation and nuclear removal, controlled by Akt, and can restore phosphorylation-defective FoxO1’s constitutive nuclear localization. The addition of a phosphate group to a protein of FoxO1 does not, however, rely on its state of acetylation [53,54]. These findings imply that the relation between the acetylation of FoxO1 and phosphorylation is not organized, where the acetylation of FoxO1 is an upward stream of phosphorylation and the phosphorylation of FoxO1 specifically represses FoxO1’s transcriptional behavior. When FoxO1 phosphorylation is disrupted [55], adipocyte differentiation is suppressed, indicating that the phosphorylation of FoxO1 is necessary for fat cell differentiation and that adipocyte differentiation can be negatively regulated by FoxO1 dephosphorylation.

In the case of non-small cell lung cancer (NSCLC) [56], FoxO1 phosphorylation facilitates cell proliferation and prevents apoptotic cell death, indicating that the nuclear barring of FoxO1 inhibits signaling pathways of programmed cell death. The phosphorylation of FoxO1 at S256 in humans dominates the phosphorylation at two other sites, namely Thr24 and Ser319, and is performed by Akt, which elevates the interaction of FoxO1 with E3 ubiquitin ligase, facilitating the nuclear transport of FoxO1 and degradation, further regulating the genetic expression and contributing to the growth, metabolism and survival of cells [57]. Alpha-lipoic acid (ALA) reduces the phosphorylation of FoxO1 in the cell HepG2, which promotes the nuclear location of FoxO1 [58] and increases the function of SIRT1 deacetylase, promoting the deacetylation of FoxO1 [59]. We, therefore, speculate that the deacetylation of FoxO1 facilitates the dephosphorylation of FoxO1 via an unidentified process that needs additional exploration.

In brief, phosphorylation-dependent nuclear removal and deacetylation-dependent nuclear retaining control the function of the transcription factor, FoxO1. By modifying the location of sub-cells, acetylation and phosphorylation coordinate FoxO1 function. Changes in oxidative damage, triggered due to the aggregation of reactive oxygen species (ROS), lead to a substantial rise in the translational function of FoxO1, which has a massive effect on the activity of superoxide dismutases (SODs) as well as catalase, which are essential anti-oxidative enzymatic proteins that are often identified as attributes or proof of oxidative damage due to the removal of ROS [60,61]. This phenomenon suggests that in the anti-oxidative signaling pathway, FoxO1 works as a sensing element.

On account of that, a study also proposed that reduced FoxO1 in several disorders, such as carcinoma-associated fibroblasts (CAFs), spindle cell lipomas, and myo-fibro-blastomas of the mammary type, may be included in the diminished transcription of enzymatic antioxidants [62].

Studies have reported O-GlcNAcylation of FoxO1 in multiple types of cell, which leads to elevation in the transcriptional activity of FoxO1. In human FoxO1, four O-GlcNAcylation sites have been recognized, however, individual mutagenesis of each site had moderate or no effect on the transcriptional activity. Furthermore, the outcome of alterations at the four sites is yet to be studied [63]. A study reported that mutation of all the four sites in mouse FoxO1 did not alleviate transcriptional activity and O-GlcNAcylation status, but tended to enhance them, which led to the recognition of a novel O-GlcNAcylated site on FoxO1, T646, whose site directed mutagenesis did not disrupt the transcription action and O-GlcNAcylation status [63].

## 3. General Pathophysiology of Obesity

Adipocytes synthesize hormones and adipokines (cell-signaling proteins). The results are affected by the distribution and quantity of fat cells found [64]. Excessive secretion within the adipose tissue of proinflammatory adipokines by adipocytes and macrophages results in a down-categorial systemic inflammatory condition in some people with obesity [65]. The hydrolytic action of triglycerides in fat cells secretes out free fatty acids, which are then transported in plasma to the areas where they can be metabolically helpful. In patients with obesity, there are increased fatty acid concentrations [66] and cholesterol levels, thus exhibiting greater fatty cell mass. Obesity accounts for increased size and volume of fat cells in the body [67].

Despite being present in fat cells, lipids are also present in various liposomes in many forms of cells [68], which are small cytoplasmic organelles near the mitochondria. Liposomes, making large cellules, promote the enlargement of liver tissue followed by a variety of pathologic conditions, such as non-alcoholic liver fat disorder, steatohepatitis, and cirrhosis [69]. An abundance of the number of lipoidal intermediates (ceramides) in certain non-fatty tissues can give rise to cell depletion and cell death [70] due to lipotoxicity. In non-adipose tissues, elevated amounts of free fatty acids, inflammatory proteins for cell signaling, and lipoidal intermediates lead to compromised insulin-resistance and insulin signaling status in many overweight or obese patients [71]. There is also a close correlation between insulin resistance and excess intra-abdominal adipose tissue [72]. Usually, a favorable ATP homeostasis continues or worsens the situation of excessive fat cell growth contributing towards obesity [73]. Large amounts of lipids spread to several body compartments with weight gain over time. At several anatomical regions that vary in physiological and metabolic features [74], subcutaneous adipose tissue retains much of the accumulated lipid.

Due to various retained triglycerides, a large number of adipocytes in subcutaneous fat cells are white; remarkably small and stable numbers of thermogenic brown and beige adipocytes are also available in adults [75]. An increase in macrophages and other immune cells in adipose tissue is followed by obesity, in particular, because of tissue modification concerning adipocyte cell death [76]. These cells of the immune system secrete out proinflammatory proteins of cell signaling, which take part in the resistance towards insulin that is frequently found in obese people.

The visceral fat cell is a lipid storage section, smaller than the subcutaneous fat cell, with omentum and mesenteric fat connected to many [77] obesity-related metabolic disorders and adverse effects. Fat cells encircle the kidney, and hypertension with nephritic compression can lead to commonly occurring hypertension in obese patients [78]. Obesity is also followed by a rise in soft pharyngeal tissues, which during sleep can restrict the airways and contribute to obstructive sleep apnea [79]. A mechanical strain on joints is also imposed by excess adiposity, making obesity a risk factor for osteoarthritis growth [80]. The increased risk of gastroesophageal reflux disease, Barrett’s esophagus, and esophageal adenocarcinoma among people who are obese [81] is allegedly due to a rise in the intra-abdominal pressure [82].

## 4. FoxO1 in General Metabolic Functions and Associated Metabolic Syndromes

### 4.1. FoxO1 in Liver

For glucose and fatty acid metabolism [83], the liver is the major organ. Glycogens contained in the liver tissues undergo hydrolysis amidst fasting to produce free glucose (glycogenolysis). Gluconeogenesis provides enough glucose after glycogen depletion by transcriptional alterations mediated by cAMP response element-binding protein (CREB), which is a gluconeogenic engine [84], controlled by transcription co-activator 2 as well as FoxO1 [85]. In the post-eating phase, the liver preserves edible carbohydrates via glycogen synthesis and de novo lipogenesis [76]. Under ordinary circumstances, the fast-feed change precisely regulates glucose production and uptake in the liver, as well as anabolic glucose release pathways, which limit post-eating amounts of plasma glucose around 4 and 7 mm [86]. A study reported a glucagon mediated FoxO1 regulatory mechanism, which further promoted stability and nuclear translocation of FoxO1 via Camp and protein kinase (PKA)-based FoxO1 phosphorylation at Ser276, which is replaced by aspartate or alanine, resulting in the blocking and mimicking of the phosphorylation of FoxO1 in liver cells of humans [87].

For genetically engineered mice [88], the crucial function of FoxO1 in liver glucose and lipoid metabolic processes is well known. Although without major modifications, in stress induction in rodents, such as extreme hunger or the removal of insulin signaling elements, a crucial aspect of hepatic FoxO1 is hard to observe firmly. By eliminating insulin receptor substrate 1 and 2 of the hepatic insulin receptor (e.g., the liver-precise Irs1 and Irs2 double knockout (DKO)-mice), the mice lack the retained insulin-regulating reflex and acquire extreme insulin receptor (IR) as well as gastro-intestinal (GI) disturbances [89]. DKO-liver transcriptome microarray shows significantly impaired development and metabolic gene expression, such as enhanced PPARγc1a (also known as Pgc1a) and Igfbp1 and reduced glucokinase, sterol-regulatory element-binding protein 1c (SREBP-c), Ghr, as well as Igf1 [90]. Furthermore, dysfunctional mitochondrial genes are downregulated as well as dysregulated remarkably after the removal of FoxO1 [91]. The insulin secretion by the activation of pancreatic β-cells intensifies the muscle and adipose [82] peripheral insulin resistance.

In DKO mice (triple knockout TKO), hepatocyte-specific knockout of FoxO1 emanates in the substantial normal functioning of the gluconeogenic genes and selective refurbishment of the fasting as well as feeding reaction, and closely regulates blood sugar and insulin levels [92]. A single mouse liver knockout of FoxO1 results in a 40% decline in energy levels at birth and a 30% decrease in adult mice after 2 days of fasting [93]. Prolonged fasting, cAMP-induced glycogenolysis, and fatty liver disease are well correlated with these modifications.

The association between FoxO1 and transcription coactivator Ppargc1a is not yet fully understood, therefore compromising the feedback mechanism of the cAMP and gluconeogenesis [94]. Likewise, the knockout of FoxO1 in the liver decreases the excessive production of glucose, triggered by liver insulin receptor ablation, avoiding diabetes in neonates and fatty liver disease in liver-precise insulin receptor overexpressed mice. These results confirm the detrimental functions of fundamentally stimulated FoxO1 throughout extreme liver insulin resistance in hyperglycemia and the insulin receptor monolayers of the insulin signaling branch phosphatidylinositol 3-kinase-protein kinase B (PI3K-Akt)-FoxO1 underlying the hepatic insulin-controlled glucose homeostasis [95].

To this extent, modified and optimized antisense oligonucleotides have been utilized to enhance both liver and peripheral insulin function for the selective suppression of FoxO1 expression (FoxO1-antisense oligonucleotide treatment) by reducing the plasma glucose amount and elementary endogenous glucose production rate in mice with diet-associated obesity [96,97]. The pathway by which IR declines to suppress gluconeogenesis, (hyperglycemia) caused by FoxO1-phosphoenolpyruvate carboxy-kinase (PEPCK), while stimulating lipogenesis induced by sterol regulatory-element binding proteins (SREBP)-1c (dyslipidemia), remains unclear.

Data from mice, lacking both primary liver controlled PI3 kinase (PIk3r1 and PIk3r2; L-p85 DKO mice), indicate that Akt and atypical protein kinase C particularly describe precise hepatic glucose and lipid metabolism activities of insulin and PI3 kinase, respectively [98]. Akt is essential for the accumulation of lipids [99] in fatty, insulin-resistant mice, as a result of the lack of leptin or rich-fatty diets. A decline in lipogenic gene activity and de novo lipogenesis is observed in the leptin-lacking obese mice with Akt, which removes hepatic triglyceride accumulation, whereas during limited hepatic Akt, mice administered with a rich-fat diet have decreased liver triglycerides with typical progression in lipogenesis, seemingly because of compensatory adjustment to dietary fat absorption. These outcomes show that Akt is a major element of insulin-regulated lipid metabolism control during IR [100].

More supporting proofs have been published recently, indicating that PI3 kinase-Akt suppression restricts both insulin-controlled PEPCK (gluconeogenesis) and [101] the gene expression of SREBP-1c (lipogenesis). The suppression of the rapamycin complex 1 (mTORC1) mammalian target, an Akt downward streaming target, can impede SREBP-1c insulin initiation but not the inhibition of PEPCK insulin, which indicates that lipogenesis and gluconeogenesis, controlled by insulin, deviate with Akt-mTORC1 pushing the prior and FoxO1. PEPCK stimulates gluconeogenesis, but FoxO tends to contribute to both [102] pathways during heavy insulin resistance, where hyperactivated FoxO leads to the aggregation of hepatic lipid [21]. Adenoviral delivery to the mouse liver of constitutively nuclear FoxO1 facilitates the aggregation of hepatic triglycerides that can advance to steatosis [21]. Reduced fatty acid oxidation is consistent with lipid accumulation. Due to FoxO1-regulated suppression of pseudo kinase tribble 3 (a regulator of Akt function) free of DNA attachment, the paradoxical activation of Akt signaling occurs. Furthermore, the blockade of FoxO1 with sufficient siRNA decreases it [103]. Corresponding research using a biologically derived nuclear FoxO1 mutant (FoxO1-ADA mutation is fundamentally nuclear because of T24 and S316 to A mutations and has an S253 to D mutation) indicates that FoxO1 switches on a feed-prospective loop relay p38 and acts to boost the behavior of Akt [104]. In conjunction, FoxO1 can trigger hepatocellular microsomal triglyceride transfer protein (MTP) expression, which controls the time-restraining stage of the attachment of triglyceride-abundant very-low-density lipoprotein (VLDL) [105]. Strengthened MTP expression is associated with elevated FoxO1 function, in correlation with increased liver VLDL output and increased plasma triglyceride concentration in transgenic FoxO1 mice. FoxO1 dysfunction triggered by the ribonucleic acid interference (RNAi)-derived liver impairment of FoxO1 mRNA lowers the development of liver MTP and VLDL in diabetic, transgenic db/db and FoxO1 mice. In control mice [106], the triggering role of FoxO1 in MTP expression and the development of VLDL is lessened by insulin.

Recent findings suggest that stimulated FoxO1 interferes with fatty acid oxidation, in contrast to the regulation of lipogenesis. By this process, under insulin-resistant circumstances [105], dyslipidemia may emerge, at least partly from mitochondrial dysfunction. FoxO1 hyper-activation in mice deficient in liver Irs1/Irs2 is followed by disrupted mitochondrial oxidative metabolic process and moderate aggregation of liver-associated lipids [91].

The transcription element FoxO1 stimulates heme oxygenase 1 by binding the promoter, which decreases the heme content necessary for the expression, stabilization, and functionality of [103] elements of the electron transport chain (ETC). In these DKO hepatocytes, the affected ETC activity reduces to oxidize NADH to NAD, thus reducing the ratio of [NAD+]/[NADH] because of the aggregation of NADH. These modifications shield two biological activities linked to the metabolism of NADH and the ratio [107] of [NAD]/[NADH]. First, the NAD-dependent deacetylase SirT1, which inhibits the PPARgc1a-mediated mitochondrial biosynthetic pathway-activation of PPARgc1a, requiring T1-catalyzed deacetylation, is inactivated. Secondly, in fatty acid oxidation, NAD serves as the critical part of acyl-CoA dehydrogenase. The removal of NADH due to ETC abnormalities attenuates the oxidation of fatty acids, which encourages the accumulation of available fatty acids for insertion into triglyceride [108]. As such, NADH oxidation pharmacological activation actively promotes the oxidation of mitochondrial fatty acids and accelerates dyslipidemia stereotactic liver in DKO and ob/ob mice [109].

**Figure 1:**
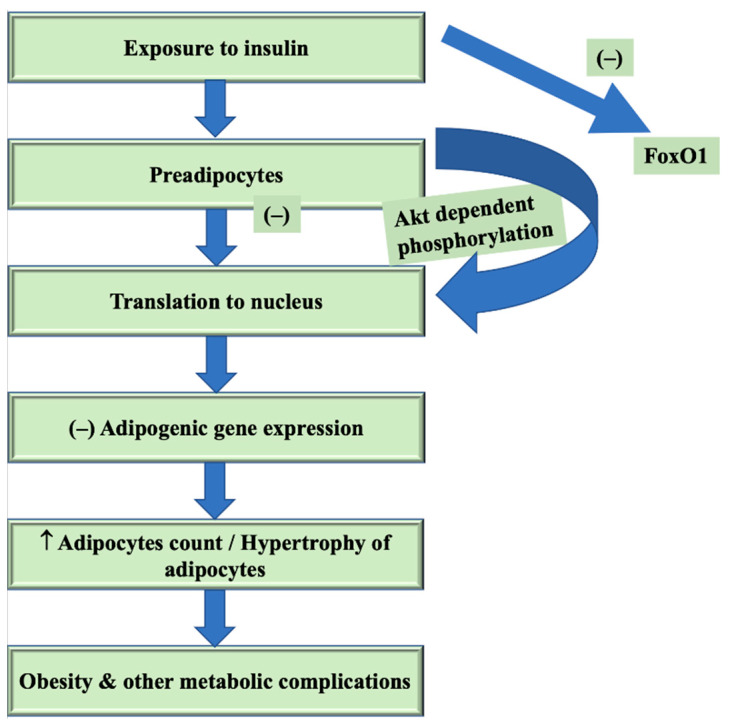
FoxO1 regulating metabolic processes and obesity. Legend: Akt—protein kinase B or PKB; FoxO1—forkhead box protein O1; (–)—inhibition; ↑—increase.

In contrast, certain findings of the adenoviral transport of fundamental nuclear FoxO1 to the mouse liver reduced fatty acid oxidation [103] show anti-sense oligonucleotide or knockout targeting hepatic FoxO1 reducing both the liver triglyceride and diacylglycerol material, which is at least partially responsible for mitochondrial function re-establishment. Together, active FoxO1 facilitates the production of hepatic glucose through gluconeogenic enzyme activation, PEPCK and 6-phosphate glucose. By modulating Akt-regulated lipogenesis via p38 and tribble homolog 3, FoxO1 can also engage in lipid metabolism. The initiation of heme oxygenase 1, which causes ETC abnormalities, concludes in the impaired oxidation of mitochondrial fatty acids and lipid aggregation under insulin-resistant circumstances [110]. Figure 1 helps in understanding the role of FoxO1 in regulating metabolic processes and obesity.

Highly functioning FoxO1 triggers hyperglycemia as well as dyslipidemia, which are the features of diabetes and diabetic problems under insulin-resistant circumstances. The concept of nutrient and metabolic homeostasis is, therefore, finely balanced by FoxO1 action [111] and controlled by FoxO1 of hepatic glucose and lipid metabolism. Feeding induces insulin release from pancreatic β-cells under normal circumstances, and FoxO1 is hindered by an insulin stimulus via the IRS-PI3K-Akt cascade [112]. The insulin signal is poor in the fasting period and FoxO1 is stimulated in the nucleus to stimulate gluconeogenesis for the supply of glucose.

However, overactive FoxO1 induces gluconeogenesis in such an unregulated manner under insulin resistance conditions that it contributes to hyperglycemia [113]. In addition, FoxO1 triggers the Hmox1 gene, which dysregulates the chain of electron transport and alters the mitochondrial metabolic pathway (including fatty acid oxidation) [114]. FoxO1 may also increase the insulin signaling downward streaming (elevating the phosphorylation of Akt) by working on Trb3 and p38, which could ultimately facilitate lipogenesis. However, when insulin refuses to trigger the IR-IRS-PIK-Akt surge, the procedure of insulin regulation in lipid synthesis falls in line with insulin-resistant circumstances [115]. FoxO1 activation is elevated in patients suffering from diabetic retinopathy by enhancing apoptotic processes in pericytes and microvascular endothelial cells. The apoptosis concentration of advanced glycation end-products (AGE) and TNF-α is enhanced in the pericytes. The mRNA FoxO1 levels, activity of DNA binding and TNF-α–mediated nuclear translocation in retinal microvascular cells are enhanced, as reported in in vivo investigations. FoxO1 knockdown by siRNA prevents the loss of microvascular endothelial cells in the retina and pericytes, which constitutes the first step in diabetic retinopathy [116].

### 4.2. FoxO1 in Skeletal Muscles

One of the main issues in the periphery accountable for insulin-driven fuel metabolic processes and glucose utilization is the skeletal muscle. Skeletal muscle accounts for about 30% of the energy expenditure at rest and 80% of the glucose uptake of the entire body [117,118]. FoxO1 expression is increased by energy distress (including fasting, calorie reduction, as well as serious diabetes) in skeletal muscles, implying that FoxO1 may facilitate the skeletal muscle’s reaction to altered energy metabolism [115].

In skeletal muscle, FoxO1 inhibits SREBP-1c, and mice over-expressing FoxO1 lack their glycemic regulation and exhibit lower physical activity ability because of extreme muscle wasting [119]. When the level of plasma glucose is low, skeletal muscle metabolism changes from the metabolism of carbohydrates to fatty acids, which are the main source for ATP production throughout the fasting period. By upregulating three enzymes, FoxO1 controls this switch: pyruvate dehydrogenase kinase-4 (PDK4), which slows down carbohydrate degradation by attacking lipoprotein lipase; pyruvate dehydrogenase (PDH), which catalyzes the hydrolytic plasma triglyceride levels in the fatty acids; and it transfers CD36, which promotes the uptake of fatty acids into skeletal muscle [120]. The catalytic process in the transition of pyruvate into acetyl-CoA can promote glucose conversion to lactate and off from acetyl-CoA [121], and PDK4 phosphorylates PDH as well as the inhibition of PDH action. FoxO1 expression increases throughout hunger or glucocorticoid therapy and specifically stimulates PDK4 activity to suppress glucose oxidation [122]. The gene activity of RXRa and SREBP-1c in the mouse skeletal muscle is turned off and on across dietary changes triggered by fasting and feeding, while FoxO1 expression shows a reverse association with SREBP-1c expression [123].

With activity, workload, or pathological disorders, such as diabetes mellitus (DM), the muscle mass of fiber size is likely to shift. A complex equilibrium of atrophy and hypertrophy [124] achieves the preservation of muscle mass. In fasting or diabetic circumstances, the stimulation of FoxO1 or FoxO3 in the skeletal muscle can enhance protein breaking via ubiquitin-proteasome and self-eating of lysosomes and the regulation of their process, constituting the two main pathways that cause muscle shrinkage [125]. Extreme scarcity can cause FoxO1-derived autophagy and atrophy, constituting the pathway that encompasses muscle wasting and sugar control under IR to dissolve protein for energy supply [126].

### 4.3. FoxO1 in Obesity

In the U.S. and other parts of the globe, the frequency of obesity has gradually risen in the past three decades. Obesity is linked with metabolic and chronic disorders including heart disease, T2DM, hypertension, and multiple types of cancer, and has put huge pressure on medical caretakers [127,128]. Obesity is a complex, multisystem disorder caused by improper lifestyle and environmental, behavioral, as well as genetic variables [129]. It is induced by an energy surplus in comparison to energy consumption due to excess calorie consumption, where the latter mainly describes a lifestyle (sedentary) and a lack of physical exercise. Other obesogenic variables also lead to the production of obesity [130], including genetic vulnerability, familial history, and gene–environment relationships.

Data indicate that the progression of obesity is linked with FoxO1. Serum concentrations of miR-27a are significantly enriched in obese liver cancer patients relative to concentrations in both non-obese liver cancer patients and normal people, where ectopically expressed miR-27a reduces the function of FoxO1 proteins in HepG2 cells. The given data reveal that the role of FoxO1 may be reduced in obese people compared to normal people [131,132]. A curious study conducted on Bamei pigs (a fatty breed) as well as Large White pigs (a skinny strain) revealed that the levels of FoxO1 mRNA and protein (*p* < 0.01) were alleviated on the first day in the subcutaneous fatty tissue of Bamei pigs and after 180 days in the fatty cells and stromal vascular parts derived from the subcutaneous fat cells of Bamei pigs, as compared to those in Large White pigs [132].

Growing research also shows that through FoxO1 [55], microRNAs (miRs) perform lipid-regulating mechanisms, which suggest that FoxO1 could be implicated in the genetic changes implicated in obesity. Elevated concentrations of acetylated/phosphorylated FoxO1 increase the function of the hypothalamic-pituitary-thyroid axis in the CNS of diet-implicated obese animals, which promotes energy consumption due to lower body weight and food intake [133]. By lowering FoxO1 acetylation and protein levels [134], SIRT1 prevents obesity caused by the insulin-unsusceptible vital nuclear location of FoxO1 in pro-opiomelanocortin (POMC) neurons in male mice. The neuro-regulation of obesity may be affected by FoxO1. Thus, this theory needs further analysis.

Overall, these findings indicate that for different therapeutic applications, FoxO1 is a promising target [131].

### 4.4. FoxO1 in Non-Alcoholic Fatty-Liver Disease (NAFLD)

Current investigations have focused on understanding the pathophysiological factors and therapies for non-alcoholic fatty liver disease (NAFLD) [135,136,137] since NAFLD is a prevalent disorder in affluent societies. Past therapeutic methods have largely targeted a decrease in weight and implementing a healthy lifestyle. A current solution, therefore, has been to resolve NAFLD co-morbid events, such as obesity, diabetes, as well as dyslipidemia [138]. Past animal studies have shown that for the induction of fatty liver disorders [139], both the quality and quantity of fat are crucial. Thus, the main goal of NAFLD therapy is to restore the metabolism of lipids. In NAFLD, FoxO1 is implicated. Prenatal exposure to ethanol substantially enhances FoxO1 expression as well as improves the vulnerability of women with intrauterine growth restriction (IUGR) offspring to NAFLD, indicating that FoxO1 could be involved in the production of NAFLD [119].

*Lycium barbarum* polysaccharides (LBPs) shield towards nonalcoholic steatohepatitis (NASH)-implicated liver damage, and LBPs counteract liver PI3K/Akt repression. These findings indicate that NAFLD is negatively affected by FoxO1. The overexpression of FoxO1 in the liver leads to a rise in triglyceride (TG) synthesis and a decline in fatty acid oxidation, aggravating liver steatosis [140]. Thus, FoxO1 possesses a vital function in NAFLD’s generation. However, SREBP1 (the more significant transcription element controlling de novo lipogenesis in the liver and a significant aspect in NAFLD pathogenesis) is negatively regulated by FoxO1, which inhibits lipogenesis, resulting in a decrease in excess fat and optimizing NAFLD therapy [141]. In addition, we believe that both functions of FOXO1 in NAFLD focus on the pathological level. Long term inquiries should validate the detailed mechanisms.

### 4.5. FoxO1 and Type 2 Diabetes Mellitus (T2DM)

T2DM, caused by peripheral insulin resistance and insufficient secretion of insulin by β cells, is now the most prevalent causative category of diabetes (>90% of case scenarios), also being defined by IR abnormalities, with impaired insulin after the predominant secretory deficiency (i.e., insulin function and secretion diseases) [142]. Thus, the function of FoxO1 as a downstream insulin factor in the development of T2DM is discussed. Pyrrolidine dithiocarbamate (PDTC) can suppress blood sugar amount and boost insulin sensitization in diabetic rodents, and Ding and colleagues stated that therapy with PDTC greatly decreases oxidative stress and induces the apoptosis of β-cells in experimentally induced T2DM rats, along with increasing insulin production by downregulating acetylation with FoxO1 [143]. These investigations show that FoxO1 could be a treatment strategy for T2DM. To facilitate FoxO1 phosphorylation, GdCl3 triggers the Akt cascade. By decreasing the expression of *Pck1* as well as *G6pc*, phosphorylated FoxO1 restricts gluconeogenesis as well as consequently reduces the cellular mass synthesis of glucose, thereby shielding towards T2DM [144]. Flavonoids defend toward T2DM by reducing the nuclear shifting of FoxO1 and the phosphorylation of Akt as well as FoxO1 in the liver and fatty tissue of rich lipid intake administered db/db mice [145]. FoxO1 also facilitates the Ang-associated anti-diabetic impact by controlling adipose triglyceride lipase (ATGL) expression. FoxO1 is also an essential factor in T2DM associated causes. In diabetic cardiomyopathy along with SIRT1-associated signaling mechanisms [146], FoxO1 also possesses a key role. Clearly, in several ways in T2DM, FoxO1 is significantly involved, indicating that FoxO1 is an essential treatment strategy for T2DM therapy [147].

## 5. Interpretation of the Network of Transcription Elements Controlling Adipogenesis

### 5.1. Fork Head-Box O Transcription Elements

FoxOs vary in their design of expression, where FoxO1 and FoxO3a are expressed pervasively, while FoxO6 exists only in the liver, although FoxO4 has not been identified in the CNS so far [148]. In the dentate gyrus, striatum, as well as ventral hippocampus, FoxO1 is primarily localized, whereas FoxO3a occurs primarily in the cortex, hippocampus and cerebellum. In the mature murine brain [149], FoxO6 is present in the amygdala, hippocampus, and cingulate cortex. Activated Akt phosphorylates FoxO transcription binding elements, which causes their nuclear exclusion [150]. This disables FoxO-mediated transcription, which under active circumstances controls cell death, development, metabolism, and cellular differentiation [151]. There are four proteins in the mammalian FoxO transcription element category: FoxO1, FoxO3a, FoxO4, and FoxO6, all of which possess a guarded DNA binding element which binds the forkhead domain (FoxHR) to the target-specific gene (G/C)(T/A)AA(C/T)AA [152] sequence of the FoxO recognized element (FRE) consensus. For instance, Fas ligand (FasL), p27KIP1 [61], as well as manganese superoxide dismutase (MnSOD) are target specific genes of FoxO1-driven transcription. FoxO1-driven transcription is regulated by post-translational alterations. The phosphorylation of various sites inside FoxOs is one significant alteration. AKT phosphorylates FoxO1 at Thr24, Ser256, and Ser319 upon activation. FoxO3a is phosphorylated via AKT [153] at Thr32, Ser253, and Ser315. In addition, based on the stimulus R, FoxOs are phosphorylated through various kinases. Ubiquitination is another post-translational alteration [154]. FOXO1 is modified via Skp2, the substrate-confined portion of the E3 ligase complex of the Skp1/culin 1/F-box protein (SCFSkp2). FoxO1 and FoxO3a are modified multiple times, whereas FoxO4 is once substituted for depletion [155]. This modification arises after the phosphorylation of FoxO1 at Ser256 through Akt [156]. FoxO transcription elements are also methylated, for example, FoxO1 is methylated at Arg248 and Arg250, which are located in the phosphorylated domain of Akt. This methylation is facilitated by the arginine N-terminal methyltransferase 1 (PRMT1) protein, which defends FoxO1 against Akt phosphorylation, nucleus transfer, and degradation [157]. Additionally, with their linked proteins such as CREB-binding protein (CBP, CREBBP) and p300-linked element, FoxOs are acetylated through CBP and p300, which reduces DNA binding and facilitates FoxO phosphorylation through AKT, which inhibits the activity of FoxOs [158]. FoxOs deacetylation is triggered by a nicotinamide-adenine-dinucleotide-(NAD) dependent histone deacetylase [159], silent information regulator 1 (SIRT1). In some systems, FoxO-mediated transcription is implicated. One of them regulates cell cycle hinderance by regulating the transcription of the cyclin-dependent kinase suppressor p27 [160]. The IR/IGF-1R signaling pathway is impaired during circumstances of growth factor deficiency and FoxOs are engaged in triggering cell cycle hinderance and quiescence to facilitate survival [13]. In contrast, the oxidative stress response includes FoxOs, which induce the occurrence of antioxidant enzymes including MnSOD [161] to combat ROS developed throughout oxidative damage conditions. Akki et al. highlighted the complex adaptive processes, constituting antioxidant systems, signal transduction mechanisms, as well as inflammatory and apoptotic pathways, which induce the protection of the cell against ROS-induced damage [162]. According to multiple in vivo and in vitro studies, when cells and tissues are exposed to low oxidative stress by environmental, physical or chemical stimulus, they exhibit greater resistance against ischemic injuries, which results in an adaptive response, referred to as ischemic preconditioning (IPC) [162], which is also triggered by FoxO1 induction, thereby retarding mitochondrial oxidative stress and improving cell survival [163].

### 5.2. PPARg and C/EBPa: Master Controller of Fat Synthesis

The function of PPARg as the primary fat synthesis moderator is confirmed by enormous evidence of both in vitro and in vivo studies [164]. Spiegelman and the workers researching for many years to illustrate the transcription elements which regulate the adipose-precise fatty acid-attaching protein aP2/FABP4 were significantly the early proofs of the critical role of PPARg in the regulation of adipogenesis [165]. This undertaking reported the recognition of an atomic element, at first known as ARF6, which later was described to match PPARg and its heterodimeric companion, RXR [166]. However, these mouse models gave only limited data on PPARg’s role in adipocytes, since each model lacked substantial evidence. In a sole scenario, the findings relied on chimeric mice obtained via homozygous embryonic stem (ES) cells [167]. The knockout cells could not become adipocytes in those species, while the wild cells formed operational adipose depots. Consequently, the effect of the absence of PPARg on adipocytes’ functioning was difficult to assess. PPARg1 is present in many tissues [168]. Published studies in pparg2/2 embryonic fibroblast mice (MEFs) have shown that ectopic PPARg1 is as appropriate for adipogenesis as PPARg2 [169]. C/EBPa is the main player of adipogenesis and has also been determined by the benefit of function analysis in cultured cells and the creation of suitable mice. Additionally, in earlier instances, Freytag and associates illustrated that uprooted C/EBPa factor could trigger adipogenesis [170] in a spectrum of fibroblastic cells. In C/EBPa impaired MEFs, PPARg can trigger adipogenesis, but C/EBPa cannot execute the adipogenic program in the vicinity of PPARg [171]. This finding indicates that C/EBPa and PPARg are involved in the individual process of adipose growth, which is primarily an adipose-related factor. It should be stated that during terminal adipogenesis, C/EBPa provides a vital role, since it failed to articulate C/EBPa and showed resistance to insulin in cell culture models, as well as failed to produce white adipose tissue (WAT) in vivo [172]. Besides the regulation of insulin action, C/EBPa was proposed to preserve PPARg expression in mature fat cells [173]. It is reported that the adipogenic phenotype of C/EBPa deficient brown adipocytes can be formed by the preservation of PPARg development using other pathways.

### 5.3. C/EBPb and C/EBPd

Many authors have classified the mechanism involved in deciding the differentiation of precursor cells into adipose tissue, far before the emergence of PPARg, as the key controller of adipogenesis [174]. It is now proven that the proliferation of transcriptional regulators ultimately gives rise to PPARg and C/EBPa expression [172]. The very first suggestion of this channel began with the work of McKnight and colleagues, which indicated that throughout adipogenesis in 3T3-L1 cells, two additional components of the C/EBP group, C/EBPb, and C/EBPd, were presented sooner than C/EBPa and that they were capable of controlling C/EBPa expression [175]. They specifically demonstrated that the lack of extracellular hormones and the ectopic activity of C/EBPb and C/EBPd in 3T3-L1 undifferentiated fat cells trigger C/EBPa expression and the fat synthesis process. They also revealed that adipogenesis can be induced, despite stimulating the expression of C/EBPa, by injecting these C/EBPs into non-adipogenic NIH 3T3 fibroblasts. However, those studies did not discuss the pathways controlling the development of PPARg [176]. Other research aimed at recognizing early adipogenesis-regulating events, which depicted a clear correlation between C/EBPs and PPARg. In particular, the uprooted expression of C/EBPb in NIH 3T3 fibroblasts, individually or in conjunction with C/EBPd, possesses activity of PPARg2 and thus promotes the transition of cells into adipocytes [172] after exposure to PPARg ligands. It has been suggested that C/EBPb and C/EBPd jointly regulate the activity of both PPARg and C/EBPa to describe the chain of events that give rise to terminal adipogenesis. Therefore, it suggests that the pathway of fat synthesis includes C/EBPb and C/EBPd initiation, thus promoting PPARg activity. PPARg then triggers the expression of C/EBPa along with these C/EBPs [177]. Still, the specific action of C/EBPb and C/EBPd in controlling the event of variables in knockout mice has been challenged, clearly showing that neonatal deficient mice, both for C/EBPb and C/EBPd, suffer from malfunction in their capacity to create fat cells. Therefore, because both variables are presented in the poorly differentiated fat cells, this alteration seems to be a downward stream of both PPARg and C/EBPa [178]. Additionally, MEFs generated through delectable mice do not possess C/EBPa or PPARg, and when collated to wild-type cells are unable to experience adipogenesis in culture [179]. These data indicate that in the previous stages of fat synthesis in vivo, where alternate mechanisms work to establish PPARg and C/EBPa expression, there is some variability. In addition, this implies that C/EBPb and C/EBPd have other roles throughout terminal adipogenesis in contrast to producing activity of PPARg and C/EBPa, since their exclusion restricts terminal fat synthesis at a phase downward stream of PPARg or C/EBPa [171]. This finding is consistent with past findings showing an ability to regulate C/EBPb expression R for cAMP signaling. In cocktails that start the adipogenic program, the demand for inducers of cAMP (isobutylmethylxanthine) is also clarified. In addition, the trigger of C/EBPd R is facilitated by glucocorticoids [180].

### 5.4. Krox20

Krox20 is a transcription factor, comprising zinc fingers, which is extensively found in the adipose tissue, but its involvement in the differentiation of fat cells has not yet been completely understood [181]. The current quantitative expression profiling of mRNAs demonstrated throughout the pre-stages of in vitro adipogenesis and in fat tissue in vivo using both microarray and qPCR analysis indicates that several specific transcription elements are possible elements of this complex system of variables involved in regulating adipogenic gene expression [182,183]. Researchers have established Krox20 as an early action factor in the adipogenic program that seems to induce C/EBPb articulation activation. Krox20 (also recognized as gene 2 or Egr2 for early growth response) is a transcription factor that is triggered instantly after cell sensitivity to mitogens. In the fat synthesis process of 3T3-L1 cells, Krox20 is triggered early and stimulates C/EBPb expression, which complies with C/EBPb to encourage terminal fat synthesis [172,184]. The fact that these pre-stages, such as Krox20, CREB, and C/EBPb initiation, initiate PPARg and C/EBPa transcription initiation by 24 to 48 h indicates that add on processes are needed to support terminal fat synthesis. In recent studies, researchers indicated that this slowdown in the operation of C/EBPb was due to the retarded phosphorylation of MAPKs and GSK3 necessary for its DNA-binding operation [185,186,187]. Other studies have also highlighted an essential phosphorylation target within the C/EBPb promoter region, but this research shows that phosphorylation controls C/EBPa expression [188], unlike Lane’s findings. More recent research indicates that the gap between both the emergence of C/EBPb and PPARg2 activity emerges from the time needed for further protein synthesis to promote C/EBPb operation.

### 5.5. KLF5 and SREBP1C

In general, the expression of the Kruppel-similar variable KLF5 is triggered by C/EBPb and C/EBPd and leads to the activation of PPARg2 [189] in combination with these C/EBPs. Neonatal heterozygous KLF5 deficient mice have a major deficit in the development of adipose tissue [190]. In contrast, in their potential to deliver adipogenesis in culture, MEFs derived from these KLF5/2 mice are impaired. Studies have also demonstrated a vital role in the progression of adipogenesis receptors for other participants of the KLF group, such as KLF6 and KLF15 [172,191]. Additional parallel pathway variables are expected to be induced initially and intersect on PPARg at a point downward streaming of C/EBPb and C/EBPd, including transcription factor helix-loop-helix (HLH) SREBP1c/ADD-1 [192,193]. A possible function for SREBP1c in controlling fat synthesis stems from various investigations, which show that in response to the insulin receptor, its activity is notably increased in 3T3-L1 fat cells. In contrast, the ectopic activity of a ruling-opposite SREBP1c has been exhibited to suppress pre-fat cell differentiation, whereas over-expression of this HLH protein greatly increases PPARg receptor adipogenic function [194,195]. Furthermore, SREBP1c expression alone is only able to induce adipogenesis to a modest degree, and further findings show that SREBP1c supports the development of PPARg [178,179,180]. In many cell types, STAT5A and STAT5B promote the transfer of cytokine release pathways to an anchor of target proteins regulating various actions [196,197]. The deletion of these STATs genes in mice contributes to many disease reactions, mainly related to the lack of growth hormone as well as the prolactin pathway that also contributes to a 5-times decrease in the mass of fat tissue relative to that of rugged animals [198,199,200]. This expression may be because of a decrease in pro-fat synthesis-prolactin, but the latest findings demonstrate that STAT5 plays a significant role in adipogenesis. In particular, the uprooted activity of STAT5A in non-adipogenic fibroblasts causes the distinction of the function of pre-adipocytes, which involves the stimulation of the role of PPARg and the aggregation of numerous fat droplets [199]. However, as the immediate ideal proteins have not yet been recognized, the mechanism underlying this behavior of STAT5A is also not understood. Human genetic experiments have been done, however, confirming STAT5’s role in controlling transcription from the pparg3 promoter [201,202]. A significant series of studies indicate that molecular biological elements may also have a role to play in controlling both the development and function of adipocytes. Krox20 is an early development reaction gene, as described above, which is triggered as convergent fat cells rejoin the cell cycle, and it also performs a significant function in producing the expression of C/EBPb and PPARg2 [203]. The in vivo investigations depicted a reduction in the basal expression of SREBP-1c mRNA in the liver by FoxO1 (constitutively active) of 60%, along with SREBP-1c induction as a response to feeding [204].

### 5.6. E2F Family

The E2F family of transcriptional regulators and accompanying pocket proteins are the most prominent cell-cycle genes, which regulate the fat synthesis process. Findings by Auwerx and associates presented suitable evidence indicating that adipocyte differentiation [205,206] is regulated by the E2F family of transcription factors. The data indicate that the opposing effects of E2F1-3 and E2F4 on differentiation tend to be due to their specific synchronization in the articulation of PPARg1. E2F4 suppresses PPARg amplification in confluent preadipocytes by interaction with the pocket protein, p130, and by recruiting the histone deacetylase HDAC3 to E2F feedback components in the PPARg1 exponent.

The enormous amount of E2F4/p130 complexes subside as fat tissue advancement through clonal expansion, while E2F1/retinoblastoma protein (Rb) complexes occur [207]. In particular, the cyclin-dependent kinase inhibitor p27KIP is a downward regulated [208,209] inhibitor which facilitates the regulation of cyclin D/Cdk4/6, which contributes to Rb phosphorylation, leading to the release of E2F1 to elicit pparg1 transcription. These data showing a role in adipogenesis for E2Fs agree with a sequence of genetic research findings in mice. In reaction to high-fat feeding, E2F12/2 mice have a restricted capacity to develop adipose tissue, while E2F42/2 ES cells lead more notably to fat tissue growth than other chimeric mouse tissues. E2F12/2 MEFs have a decreased ability to distinguish into fat cells, consistent with mouse models, while E2F4-lacking MEFs and ES cells show an enhanced ability to differentiate.

In addition, the simultaneous loss of the main pocket proteins p107 and p130 correlated with E2F4 causes greater fat synthesis inconsistent with MEFs [210,211], supporting the notion that pparg1 transcription is repressed by E2F4/p107 or E2F4/p130 combinations and not E2F4 itself. A deficiency in Rb would be predicted to improve fat synthesis by simplifying the action of E2F1, but Rb2/2 MEFs have an interestingly decreased capacity for differentiation into white adipocytes [209,212,213].

**Figure 2:**
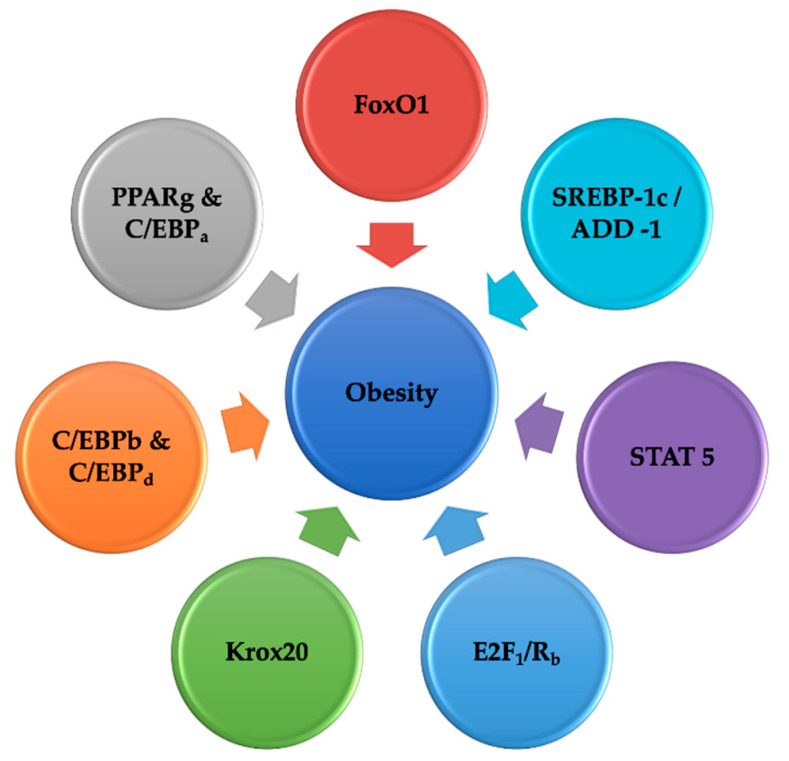
Transcription factors and transporters regulating obesity. Legend: PPARg—Peroxisome Proliferator Activated Receptor Gamma; C/EBP—gene; SREBP1—sterol regulatory element-binding protein 1; Krox20—member of early growth response (EGR) genes; E2F—retinoblastoma binding to E2 transcription factor; STAT 5—Signal transducer and activator of transcription 5; FoxO1-forkhead box protein O1; C/EBPb—enhancer-binding protein beta; C/EBPd—enhancer-binding protein delta; C/EBPa–CCAAT enhancer-binding protein alpha.

This is possibly due to the need for Rb to promote cell-cycle escape, and also cooperation to trigger adipogenic gene expression with C/EBPs. Therefore, it also seems that two distinct but parallel paths that conclude in the stimulation of PPARg1 and PPARg2 activities are regulated by the E2Fs and pocket proteins. Precisely, components including Rb channeling via C/EBPb give rise to the synthesis of PPARg2, while elements promoting the activity of E2F1 give rise to the activity of PPARg1. Since the predominant regulator of adipogenesis is considered PPARg2 and not PPARg1, components including E2F that converge on PPARg1 require different ways of elevating the activity of PPARg2. This system could be aided via C/EBPa, but PPARg1 stimulates C/EBPa which stimulates PPARg2 activity. These procedures help in understanding redundant information in pathways regulating PPARg2 articulation in mice lacking in C/EBPb and C/EBPd. In such mice, it is plausible that instinct in the emerging fat storage action on E2F to enhance PPARg1 thus stimulates C/EBPa led by PPARg2 independently for activity of C/EBPb or C/EBPd [214]. Moreover, E2f1 and FoxO3 are the two transcription factors which have been reported to play a significant role in cellular senescence [215]. Therefore, some transcriptional factors are involved in the genetic regulation of obesity (Figure 2).

## 6. FoxO1 in Pathogenesis of Obesity

The transcriptional activators regulate the hypertrophy of adipocytes. The slight difference in the behavior of favorable versus detrimental effectors is likely to decide whether or not adipogenesis would continue within a specific progenitor cell population. The distinction of mesenchymal stem cells into fat cells is inhibited [214,216] by activating the Wnt signaling pathway, which unlike adipocytes [217], tends to favor the division of procreator cells into bone or muscle. Wnts are a broad class of exogenic responses released during early development by several cell types, and they perform a deciding activity. The coupling of different Wnts to subsequent Frizzled receptors and proteins linked to low-density lipoprotein receptors (LRPs) triggers signaling pathways, which modify gene expression and function of cells.

The orthodox Wnt processes contribute to β-catenin mobilization towards the nucleus, thus, transcription elements of the TCF/LEF family are coactivated. The vulnerability of premature fat cells to Wnts or uprooted activity of a fundamentally active form of β-catenin prevents fat synthesis by suppressing PPARg and C/EBPa induction [217,218]. The exact mechanisms implicated are not understood, but they possibly incorporate the expression of target genes of TCF/LEF since supreme opposite TCF (dnTCF) expression partly releases Wnt suppression actions. Cyclin D1, which is directly involved in Wnt signaling, opposing PPARg effects [209,219], is an appealing candidate for a TCF-induced adipogenic inhibitor. It is also conceivable that β-catenin could impart the inhibitory actions of PPARg by pathways other than those comprising TCF/LEF [220], and it should be noted that the conditional removal of β-catenin in the emerging mouse mesenchyme results in the switching of myometrium [221,222] adipogenesis. Several experiments have shown that by compromising C/function, EBPb’s numerous effectors attenuate adipogenesis. Not only do these findings recognize the presence of suppressors, but they also affirm action for C/EBPb in controlling the distinction of preadipocytes. A number of these suppressors are found in preadipocytes, namely ETO/MTG8, GATA2/3, GILZ, CHOP10, and delta interacting protein A (DIPA), and their expression throughout differentiation is downward regulated.

It is important to remember that the vitamin D receptor, which is stimulated at first in adipogenesis [223,224], suppresses the differentiation of preadipocytes by downregulating C/EBPb via pathways that may include ETO/MTG8 activation [225,226]. Similarly, Hedgehog signaling, which is believed to control the progression of vertebrates, performs a conserved role in suppressing the development of fat, likely by triggering GATA2 expression [227,228,229]. Other researchers hypothesized that by controlling adipogenesis, oxygen tension could regulate adipose tissue development.

Precisely, Yun et al. (2002) have shown that oxygen deficiency prevents the distinction of preadipocytes via a process that includes DEC1/Stra13 suppression of PPARg activity. DEC1/Stra13 is a component of the split transcription repressor family of Drosophila hairy/Enhancer, caused by oxygen deficiency-instigated replication factor 1a. Stra13 is also caused by retinoic acid (RA) [230,231], and may therefore also be the moderator by which adipogenesis is inhibited by RA. Insulin has considerable proadipogenic activity, as mentioned above, in part by facilitating SREBP1c expression. Studies conducted in both animals and cell lines show that insulin enhances fat synthesis by blocking the FoxO1 suppressor activity. Explicitly, the manifestation of preadipocytes to insulin gives rise to the phosphorylation of FoxO1 depending on Akt, shielding its translocation to the nucleus and subsequently inhibiting the expression of the adipogenic gene [231,232].

Accili and associates have shown that consistently active FoxO1, which is inconsiderate to Akt phosphorylation, interrupts the division of 3T3-F422A pre-mature fat cells during the replication of clones to recognize factors involved in this inhibitory behavior [233,234]. This suppression in the adipogenic development is probably due to the initiation of the cyclin-dependent kinase inhibitor p21CIP R correlated with FoxO1. Subsequent studies have shown that FoxO1 haploinsufficiency (FoxO1/2) defends diet-induced insulin resistance/diabetes by restricting the increase in the size of fat cells [234] to promote the inhibitory effect of FoxO1 in adipose tissue.

**Figure 3:**
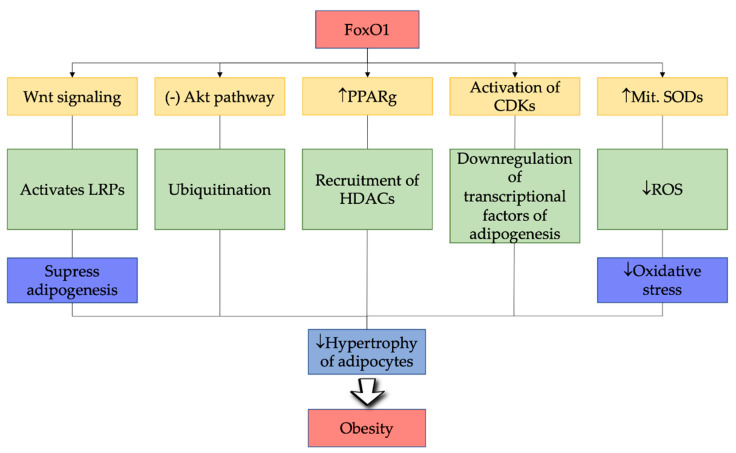
Mechanistic approaches of FOXO1 in obesity. Legend: FoxO1—forkhead box protein O1; PPARg—peroxisome proliferator-activated receptor gamma; CDKs—cyclin-dependent kinases; ROS—reactive oxygen species; SODs—superoxide dismutases; HDACs—histone deacetylases; LRPs—laterized readiness potential; ↑— increase; ↓—decrease.

It is noteworthy that adipogenesis upstream of PPARg is also attenuated by two additional participants of the forkhead family, FoxA2 and FoxC2. It is worthwhile to note that although three subtypes of the KLF group are more prone to fat synthetic processes (KLF5, KLF6, and KLF15), at least one KLF serves as an adipogenesis inhibitor. In particular, in pre-adipocytes, the KLF2/lung Kruppel-similar element is shown primarily in adipose tissue, and its expression throughout adipogenesis [190] is downregulated. The pparg2 transcription is inhibited by the ectopic activity of KLF2 in preadipocytes, likely by coupling to KLF controlling key factors in the same section of pparg2 that promotes the previous fat synthetic processes and action of KLF5 [190,191]. The review thus describes the role of FoxO1 in regulating the pathogenic pathways involved in obesity, as mentioned in Figure 3.

## 7. Conclusions

FoxO1 seems to participate in the management of anti-oxidative stress, metabolic processes, and other physiological and pathophysiological methods. FoxO1’s function in its target elements suggests that if we could certainly explain its regulatory activity, FoxO1 could be an emerging optimized pathway for targeted treatment. It is worth mentioning that it is unknown if all FoxO1 targets are regulated during one form of illness. It is uncertain if this inconsistency arises from the disparity in the degree and length of therapy. As a long-life component of an individual, whether FoxO1 smartly picks the multiple targets to prevent distress during the adjustment cycle/decompensation period still requires investigation. Besides, FoxO1 acts as a vertex of many signaling coordinates and feedback to different burdens. For the progress of the disease, equilibrium in the intracellular transcriptional role of both FoxO1 and its modulators is important, while the mechanistic pathway is not completely determined, being obvious that the post-translational modifications of FoxO1 need to be further explored. In conclusion, sequential exploration and descriptions for the identification of successful therapeutics that may be categorized in novel disease therapy pathways await the mechanism of the precise control and application of FoxO1.
